# Case Report: Can this 90-year-old patient with cT4N3M0 NSCLC have concurrent chemoradiation for cure? Learning from a representive case

**DOI:** 10.3389/fonc.2026.1896102

**Published:** 2026-09-11

**Authors:** Cuimeng Tian, Jinliang Zhang, Wenji Pu, Shing Cheung Mathew Yik, Biaoda Li, Feng-Ming (Spring) Kong

**Affiliations:** 1 Department of Radiation Oncology, Beijing Chest Hospital, Capital Medical University/Beijing Tuberculosis and Thoracic Tumor Research Institute, Beijing, China; 2 Department of Thoracic Oncology, Clinical Oncology Center, The University of Hong Kong-Shenzhen Hospital, Shenzhen, China; 3 Department of Radiation Oncology, Shenzhen Nanshan People's Hospital, Shenzhen, China; 4 Department of Clinical Oncology, Tuen Mun Hospital, Hong Kong, Hong Kong SAR, China; 5 Department of Radiology, The University of Hong Kong-Shenzhen Hospital, Shenzhen, China; 6 Department of Radiotherapy, Clinical Oncology Center, The University of Hong Kong-Shenzhen Hospital, Shenzhen, China; 7 Department of Clinical Oncology, Li Ka Shing Faculty of Medicine, The University of Hong Kong, Hong Kong, Hong Kong SAR, China; 8 Departments of Clinical Oncology, The University of Hong Kong-Shenzhen Hospital and Queen Mary Hospital, Li Ka Shing Faculty of Medicine, The University of Hong Kong, Hong Kong, Hong Kong SAR, China

**Keywords:** 90-year-old, case report, concurrent chemoradiation, cT4N3M0 NSCLC, adaptive radiotherapy

## Abstract

It is challenging to manage locally advanced non-small cell lung cancer (NSCLC) in patients older than 85 years, as there is neither a guideline nor evidence for guidance. Most clinical trials excluded this population for enrollment. Here we report a case of a 90-year-old former smoker with squamous cell carcinoma of the left upper lung, FDG-PET/CT staged IIIC cT4N3M0 (the 8th Edition of the UICC/AJCC Staging System). The patient, representing the population of older patients, was remarkable for extensive vertebrae body invasion with the primary tumor abutting the true spinal cord. Our multidisciplinary team consultation rendered him with unresectable tumors in a medically inoperable condition. The patient was treated with concurrent chemoradiation therapy to a tumor dose of 60 Gy in 30 fractions, using an imaging-guided adaptive technique to keep the cord in a safe dose limit, similar to that of RTOG1106. The patient had quick and lasting pain relief and tumor shrinkage during the course of therapy, which allowed adaptive cord-sparing therapy. The patient completed the definitive course of chemoradiation with Grade I/II radiation esophagitis and Grade IV neutropenic fever. He did not have subsequent immunotherapy maintenance. He is now almost 6 years out from the lung cancer diagnosis, with excellent performance status without any evidence of disease progression. His tumor continuously shrunk 3 years after the completion of radiotherapy; the destroyed bone recovered to a normal appearance. He has no evidence of disease progression nor any notable side effects. The success of this case implicates that the curative potential of concurrent chemotherapy with precision personalized adaptive therapy alone without adjuvant immunotherapy in the extremely old population. Future prospective studies are needed.

## Graphical Abstract

**Figure:**
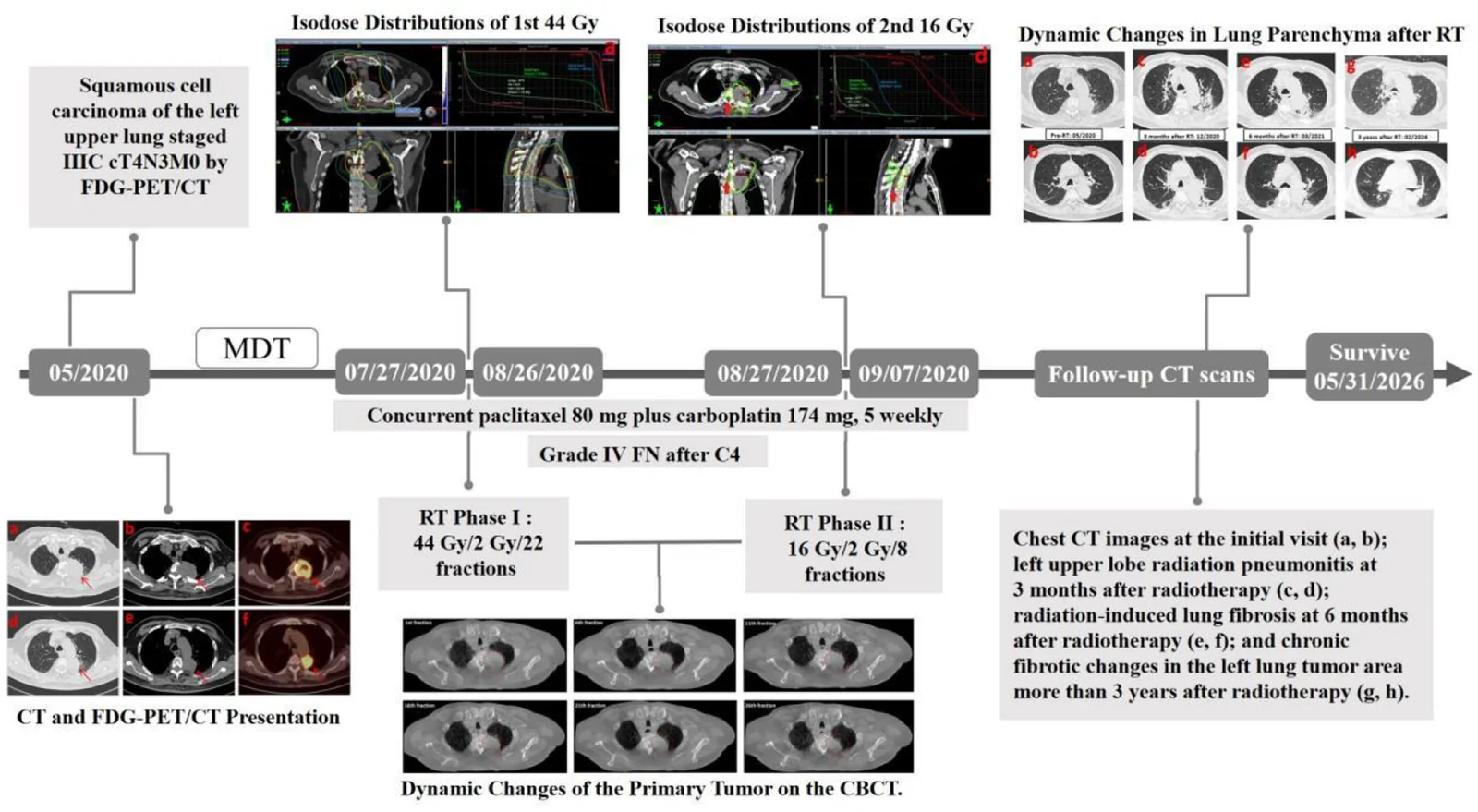

## Introduction

It is unknown whether extremely old patients would equally benefit from aggressive curative regimen for management. Most physicians would offer palliative approaches; some will weigh the benefits and risks before deciding on definitive aggressive therapy. Here we report management and results of a 90-year-old patient with locally advanced non-small cell lung cancer (NSCLC).

## Case report

### Patient information

**Figure 1:**
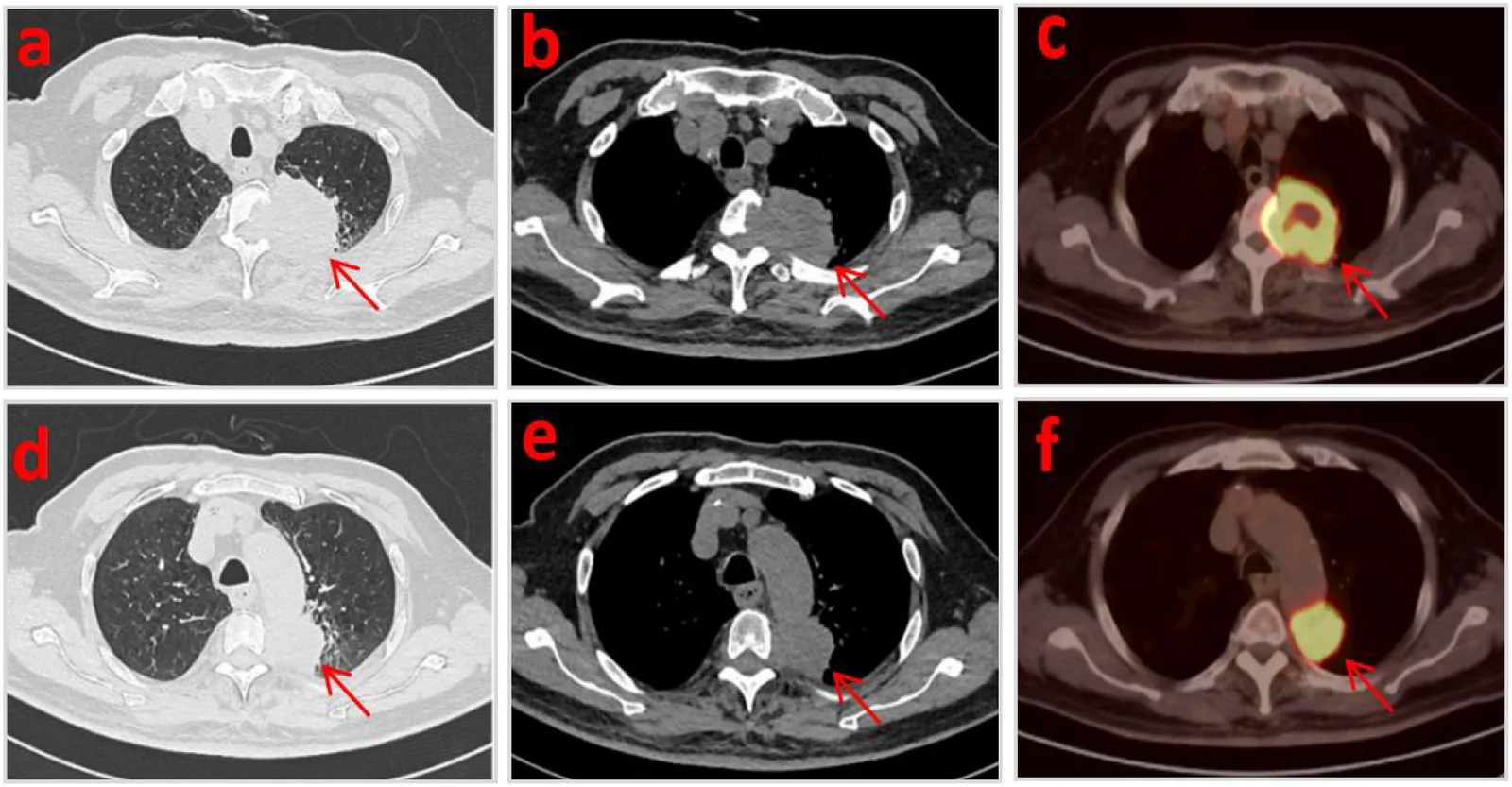
CT and FDG-PET/CT presentation of a T4 left upper lung cancer. The image shows a tumor from the upper left lung under lung window **(a, d)** mediastinum window **(b, e)** and FDG-avid on PET-CT **(c, f)**. CT scans **(a–c)** demonstrate extensive invasion of the adjacent vertebrae bodies with tumors occupying almost 50% of a vertebrae body.

This is a 90-year-old gentleman, a former smoker, who presented with severe progressively worsening back pain and was subsequently diagnosed with squamous cell carcinoma of the left upper lung, FDGPET/CT-staged IIIC cT4N3M0. He sought medical care in May 2020. A chest CT scan with IV contrast revealed a 5.1cm×3.9cm mass in the posterior segment of the left superior lobe tip, adjacent to the left 3rd posterior rib and the 2nd and 3rd thoracic vertebrae. There were multiple lymph nodes in the mediastinum 2L, 4R/L, Zone 5, and the left upper hilus of the lung, with the larger one measuring about 1.4cm×1.0cm, suspected to have metastasized lymph nodes. A PET-CT scan showed a hypermetabolic lesion near the mediastinum in the posterior segment of the upper lobe tip of the left lung, which was considered to be a malignant tumor with adjacent bone invasion and obstructive pneumonia (**Figure 1**). Multiple slightly enlarged lymph nodes in the mediastinum showed limited FDG uptake, with one node in the right level 4 concerning metastasis Pathology from a CT-guided left upper lung mass biopsy on May 28, 2020, reported squamous cell carcinoma. Next genome sequence testing revealed no driver mutation from the tumor tissue, TMB 8.24/hf, microsatellite stable, PD-L1 <1%. A combined assessment of CT and PET/CT rendered this patient as having cT4N3M0, stage IIIC. The patient was otherwise healthy with no major comorbidity.

The patient sought medical attention due to severe chest and back pain, predominantly confined to bed rest, with an ECOG performance status score of 1. Baseline pulmonary function tests showed: FEV_1_ 1.56 L, FEV_1_ 49% of predicted value, FVC 1.62 L, and FEV_1_/FVC ratio of 65%. All routine blood parameters were within normal limits. Among liver and kidney function tests, serum uric acid was elevated at 483 μmol/L (reference range: 202–416 μmol/L), while the remaining parameters were within normal ranges. The patient had a chronic history of hypertension and hyperlipidemia for over 25 years, for which he had been taking oral antihypertensive agents and statins.

### Treatment received

The patient was already seen in several outside hospitals and was recommended to have palliative care for pain relief only. The patient and family were interested in any possible curative measures. He was discussed in our multidisciplinary team (MDT) meeting and was not considered to be a candidate for surgical treatment due to his age and the extensive invasion of the thoracic vertebral bodies. Thoracic radiotherapy plus minus concurrent chemotherapy(CCRT) and adjuvant immunotherapy was considered. There was active discussion on possibilities of giving a curative dose versus a palliative dose of radiotherapy due to the proximity of the tumors to the spinal cord. A mid-treatment tumor-adaptive spinal cord-preserving strategy was planned.

**Figure 2:**
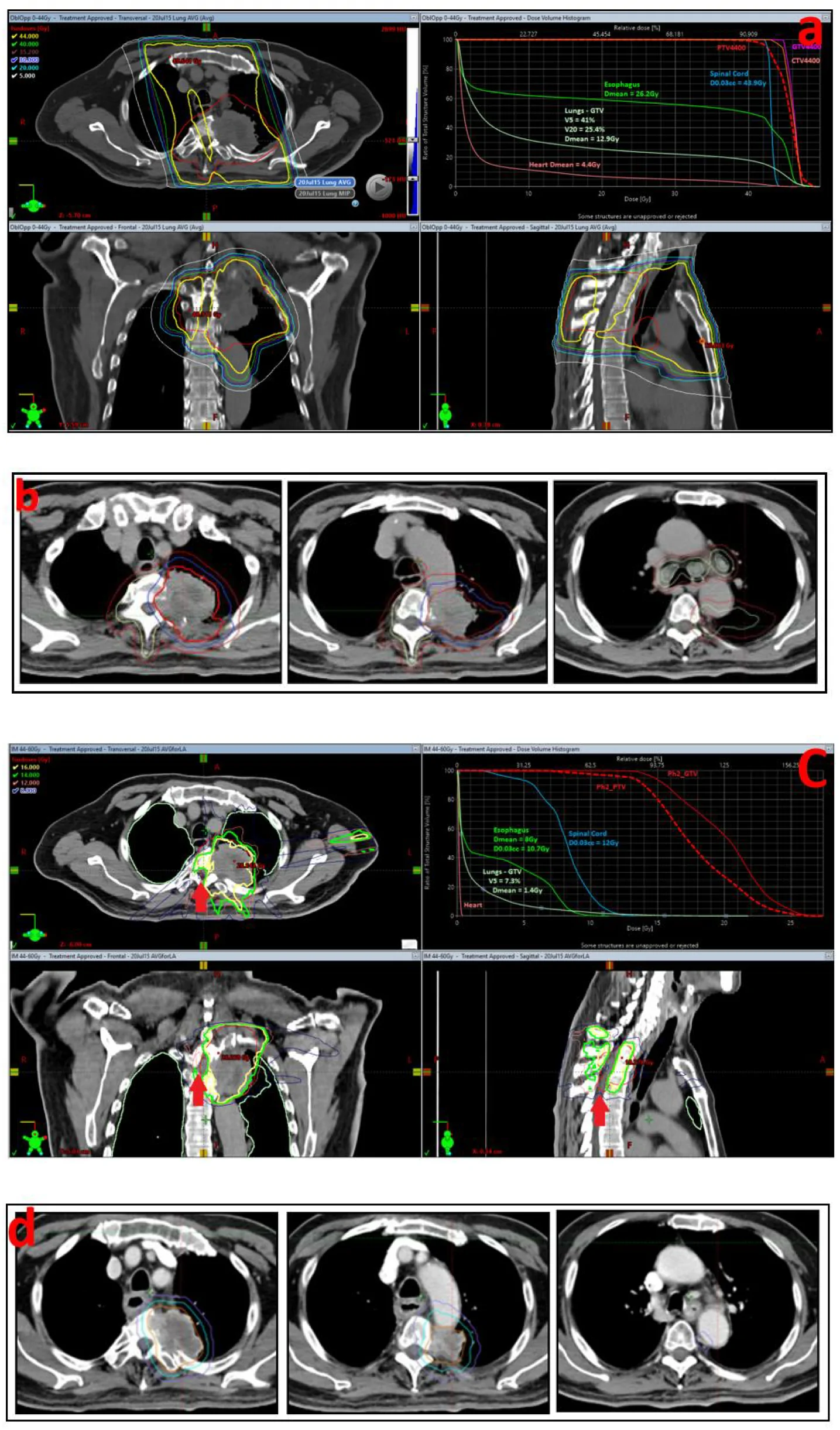
Isodose distributions of 1st 44 Gy and the adaptive radiotherapy plan. The panel **(a)** shows the radiation dose distribution of the first 44 Gy, on transverse, coronal, and sagittal views. The Phase I Clinical Target Volume (CTV) included the left upper lung tumors with a 5 mm margin from GTV, tumor involved vertebral bodies, level 4R/L lower paratracheal the mildly FDG avid nodes, level 5 a/p window node, and left level 10 hilar nodes **(b)**. The isodose distribution of the adaptive phase shows adaptive boost dose on the primary tumor **(c)** and the Phase II CTV of the adaptive radiotherapy target included only the left upper gross lung tumors with a 5 mm uniform margin **(d)**, without inclusion of the nodes.

**Figure 3:**
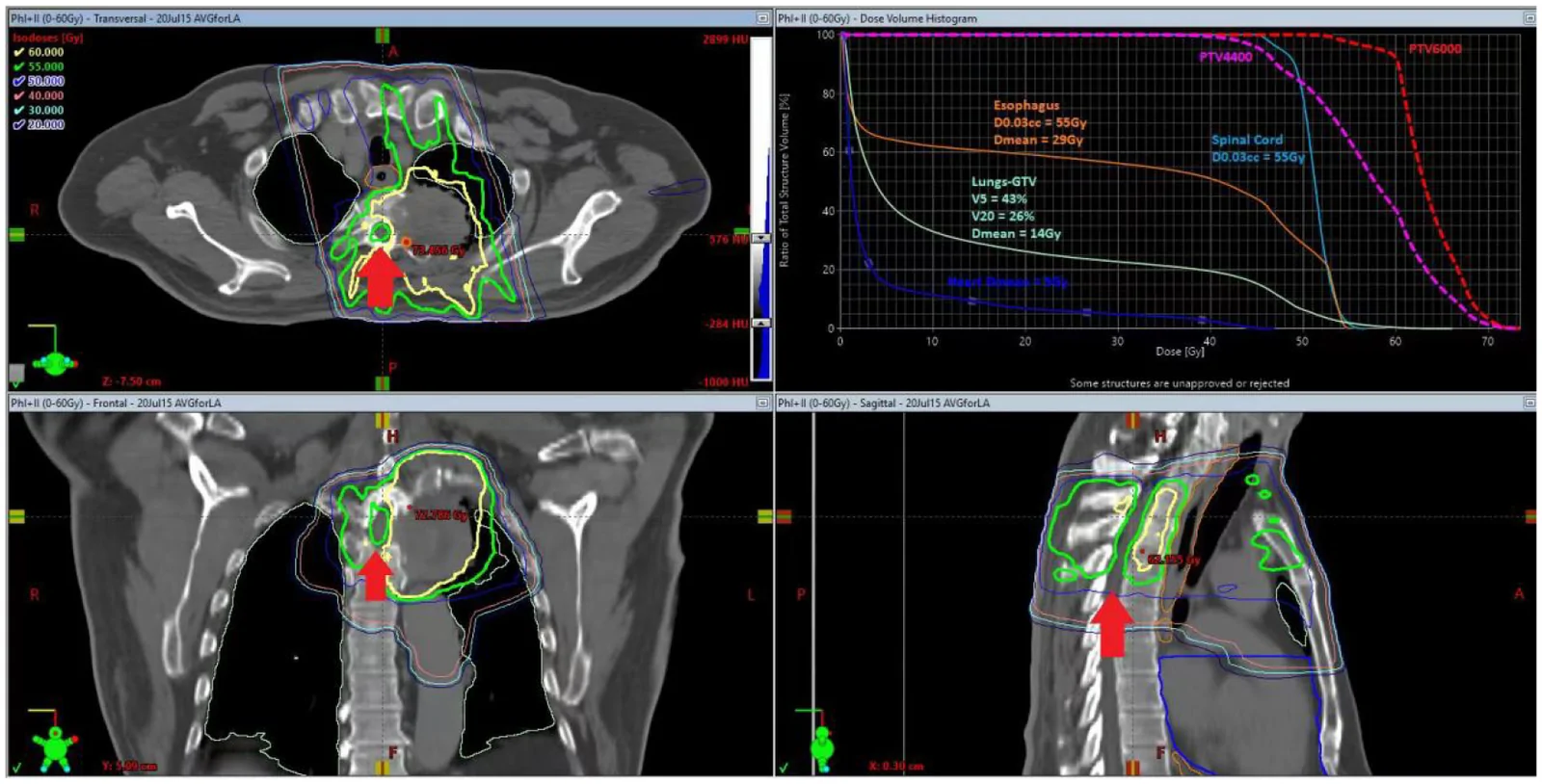
Isodose distribution of the full course radiotherapy. These scans show radiation isodose distributions of the composite plan of phase I and phase II for a total dose of 60 Gy to the planning target volume. The mean lung dose to both lungs minus GTV was 15 Gy, and the maximum cord dose (dose to 0.03 cc) was 55 Gy.

Radiotherapy received: The prescription dose of radiotherapy was 60 Gy delivered over 30 fractions using adaptive planning mid-treatment, a planning strategy similar to that of RTOG1106 (1). Phase I consisted of 44 Gy administered over 22 fractions from July 27, 2020, to August 26, 2020. Target and OAR dosimetrics: maximum dose to spinal cord PRV 45.8 Gy, mean heart dose 4.4 Gy, maximum esophageal dose 48.3 Gy, mean lungs-GTV 12.9 Gy (V5 41.0%, V20 25.4%) (**Figure 2**). Phase II involved 16 Gy delivered over 8 fractions between August 27, 2020, and September 7, 2020. Target and OAR dosimetrics: maximum spinal cord PRV 21.1 Gy, mean heart dose 0.1 Gy, maximum esophageal dose 11.4 Gy, mean lungs-GTV 1.4 Gy (V5 7.3%, V20 0.0%), and maximum true cord dose 21.0 Gy (**Figure 3**). The composite maximum dose to the true spinal cord was 55 Gy/30 fractions(physical dose); the mean lung dose was 14 Gy. A total of 5 weekly chemotherapy with paclitaxel 80mg plus carboplatin 174 mg were administered concurrently during the course of radiotherapy(The weekly concurrent chemoradiotherapy regimen was calculated based on a body surface area of 1.96 m^2^ in accordance with the NCCN Guidelines. Carboplatin AUC 2).

The gross tumor volume (GTVp) was 158.3 cc before RT, shrunk to 112.2 cc after 44 Gy of RT, resulting in a 29% volume decrease. There were no significant changes in lung parenchyma on the during-treatment CT scan.

### Treatment response and long-term outcome

**Figure 4:**
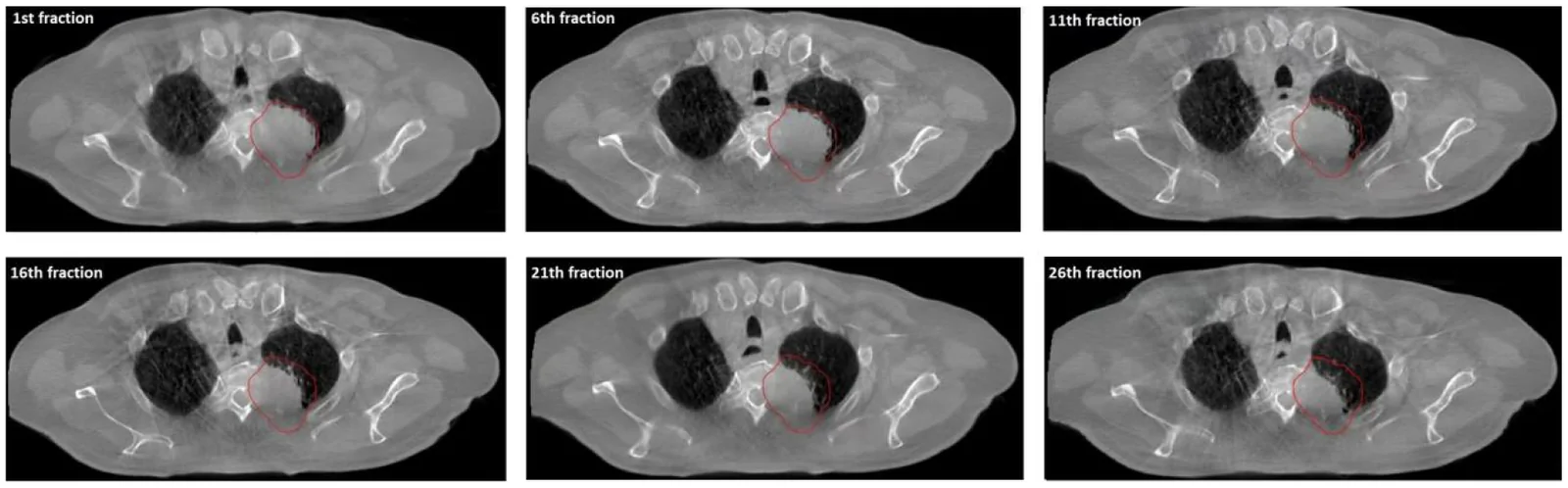
Dynamic changes of the primary tumor on the CBCT. The picture shows a gradual course of primary tumor shrinkage from baseline at 1^st^, 6, 11, 16, 21, and 26 fractions from the radiotherapy start. Redline shows the primary tumor at baseline.

The patient had a good immediate response to treatment, had pain decreased, and eventually resolved at the end of the treatment, consistent with tumor shrinkage shown on weekly CBCT (**Figure 4**).

**Figure 5:**
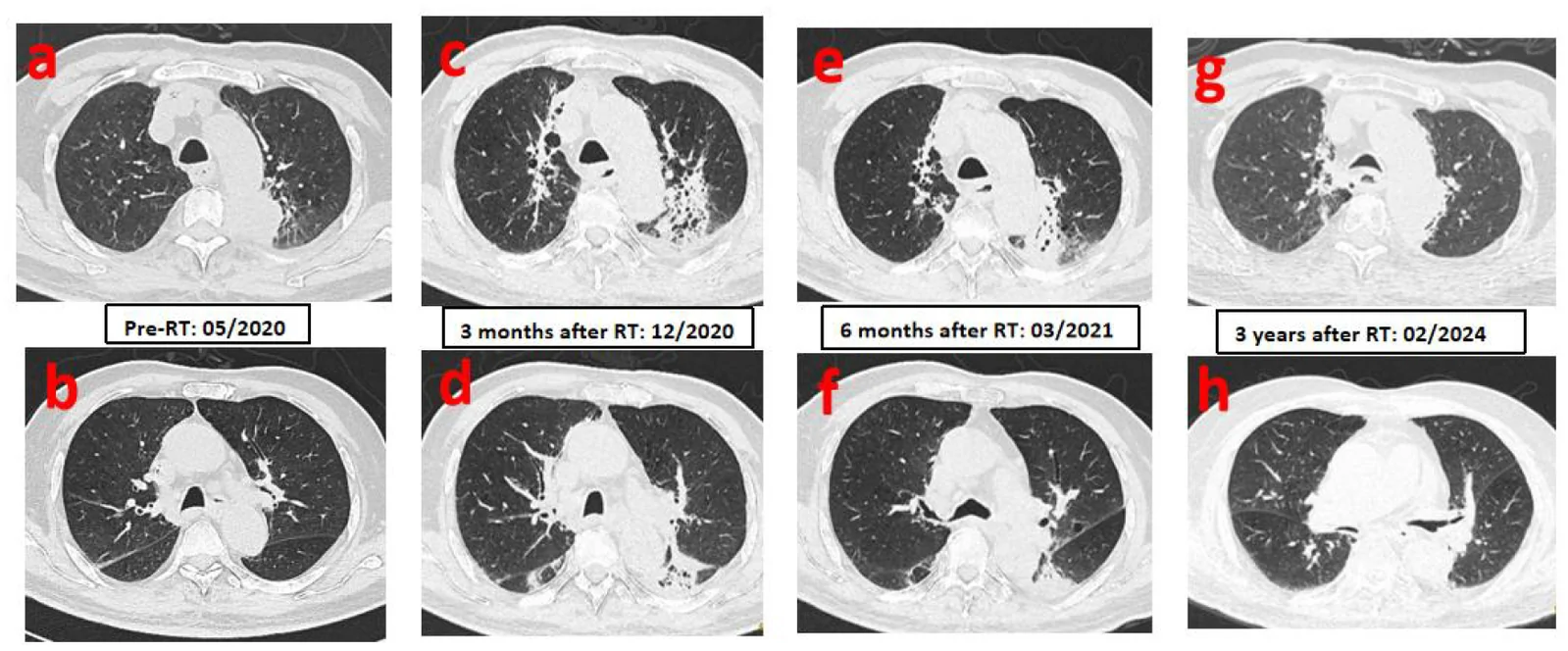
Dynamic changes in lung parenchyma after radiotherapy. Follow-up CT scans were shown after radiotherapy: Chest CT images at the initial visit **(a, b)**; left upper lobe radiation pneumonitis at 3 months after radiotherapy **(c, d)**; radiation-induced lung fibrosis at 6 months after radiotherapy **(e, f)**; and chronic fibrotic changes in the left lung tumor area more than 3 years after radiotherapy **(g, h)**.

**Figure 6:**
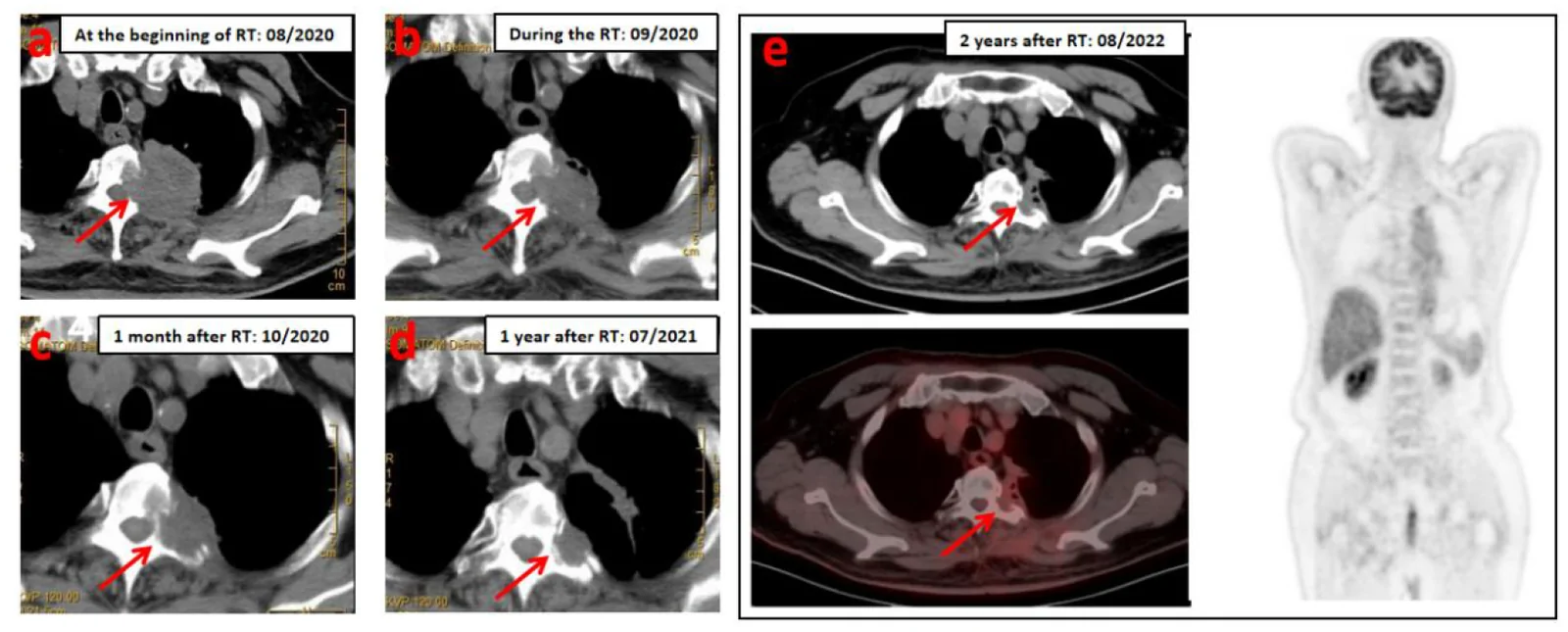
Dynamic changes of tumor-invaded vertebrae bone after radiotherapy. There is extensive destruction of the tumor-invaded vertebrae at the beginning of **(a)**, during **(b)**, 1 month **(c)** and 1 year **(d)** after radiotherapy (RT) and slow but gradual shrinkage of the destroyed bone to almost normal appearance at 2 years **(e)**. At two years after RT, the PET-CT shows minimal residual tumor, without remarkable metabolic activity of the tumor.

The patient completed the above definitive course of chemoradiation with Grade IV neutropenic fever after the forth cycle of chemotherapy and Grade I/II radiation esophagitis starting at the 3rd week of treatment. The Grade IV febrile neutropenia resolved after treatment with recombinant human granulocyte colony-stimulating factor and meropenem. Prophylactic G-CSF was administered following the fifth cycle of chemotherapy, which was delivered at the full planned dose. The esophagitis symptoms were managed by topical and medications. His pain was completely resolved by the end of the treatment. His dysphagia was managed conservatively and resolved in 2 weeks after completion of radiation. He did not receive any adjuvant therapy, as the preference of the patient and family and presence of radiographic lung inflammations, and the MDT’s risk-benefit assessment, which raised concerns regarding immune-related adverse events in the very elderly and the uncertain benefit of immunotherapy in immunosenescent populations. He has been followed up regularly in the clinic. Follow-up chest CT scans showed reduced sizes of the tumor, destructed bone structures, and the presence of fibrosis around the primary tumor site. The grade I (radiographic) radiation pneumonitis, was resolved without any treatment (**Figures 5**-**6**). He is now almost 6 years out from his lung cancer diagnosis, functioning very well with no evidence of disease progression and without any side effects.

## Discussions

Can a patient with advanced NSCLC of 90 year-old tolerated and benefit from an aggressive and curative treatment? The standard treatment for patients with unresectable locally advanced NSCLC had been CCRT until the report of the Pacific Study (2). Data for elderly patients, however, is limited. In clinical practice, CCRT is approached with caution for older individuals due to concerns about their ability to tolerate the aggressive treatment and potential increased risk of side effects. Treatment decision for older patients with locally advanced NSCLC often involves a careful assessment of their overall health status, comorbidities, and functional status to determine the most appropriate treatment approach that balances the potential benefits and risks. Based on a randomized controlled trial conducted by the Japan Clinical Oncology Group (JCOG0301), low-dose carboplatin and concurrent thoracic radiotherapy have been established as the standard treatment for elderly patients with unresectable locally advanced NSCLC. In that study, the median overall survival time for the chemoradiotherapy group was 22.4 months. This treatment approach aims to provide effective therapy while minimizing the potential for treatment-related toxicity in older individuals (3). In a phase I study, the combination of carboplatin/nab-paclitaxel and concurrent thoracic radiotherapy demonstrated good tolerability and showed promising efficacy in elderly patients with locally advanced NSCLC (4). However, it is important to note that the oldest patients enrolled in the study were 86 years old, indicating that evidence was lacking when considering this treatment approach for very elderly individuals. Indeed, the highest age reported in published clinical trials for elderly, inoperable, stage III non-small cell lung cancer was 83 to 86 years old (5–12), as listed in **Table 1**. Success in this case of 90 years suggests the age cut-off may not be appropriate for all. Further clinical trials should individualize the age consideration; maybe it is better not to set an age limit. This real-world example demonstrates that a curative approach may be feasible and effective even in older patients, highlighting the importance of individualized treatment decisions based on the patient’s overall health status and treatment tolerance. It is essential to consider the potential benefits and risks of treatment in elderly patients with locally advanced NSCLC to optimize outcomes while minimizing adverse effects.

**Table 1:** The age criteria for elderly patients with locally advanced unresectable stage III non-small cell lung cancer in clinical trials. .

Study	Research type	Age range, years	Maximum age, years	Treatment plan
RTOG 0617 (12)	a randomized, two-by-two factorial phase 3 study	37-83	83	radiotherapy with concurrent and consolidation carboplatin plus paclitaxel with or without cetuximab
RTOG 1106 (11)	A Phase 2 Clinical Trial	45-83	83	Midtreatment PET/CT-Adapted Radiation Therapy With Concurrent Chemotherapy
PACIFIC-6 (8)	The Phase 2 Trial	39-85	85	durvalumab after sequential CRT
SPRINT (5)	prospective study	53-86	86	sequential pembrolizumab and risk-adapted radiotherapy
DOLPHIN (6)	Phase 2 Nonrandomized Controlled Trial	44-83	83	radiotherapy in combination with concurrent and maintenance durvalumab immunotherapy
Zhou Y M (7)	retrospective study	47-83	83	Four-phase split-course chemotherapy and radiation
Zaborowska-S M (9)	retrospective study	53- 68	68	sequential chemoradiotherapy
Niho S, Hosomi Y (10)	a multicenter Phase I/II study	71-83	83	Carboplatin, S-1 and concurrent thoracic radiotherapy

Can patients with stage III NSCLC without driver mutation be cured without adjuvant immunotherapy? This is another important question, which is highlighted by this case of a cure without adjuvant therapy. The PACIFIC study (2) has set up the standard of care for stage III NSCLC as adjuvant immunotherapy, which provided significantly better results. One has to note, however, that patients in the placebo group without adjuvant immunotherapy had a 5-year overall survival rate of 33.4%, indicating that these patients did not benefit from adjuvant immunotherapy. Who could be cured with immunotherapy is a question of importance for personalized precision therapy. The expression level of PD-L1 is highly correlated with the treatment response; those with a high level of expression would benefit more significantly from immunotherapy. Microresidual disease detection by ctDNA may help, but data is limited in this setting. Success of this case without adjuvant immunotherapy cannot devalue this role. It was suggested that the efficacy of immunotherapy may be related to age. A retrospective study reported that the median OS of immunotherapy in patients aged 80 or above was only 3.62 months (13). A further unresolved issue is the potential correlation between older age and immunotherapy primary resistance, a phenomenon probably linked to the continuous and progressive remodeling of immune functions with aging, known as immunosenescence (14). Exploring the molecular and immune mechanisms underlying a potential lack of benefit or even accelerated tumor growth during immunotherapy in elderly patients represents a new direction for future research in this field of cancer treatment.

It is also worth discussion regarding the radiation technique applied for a T4 tumor with the primary tumors abutting the spinal cord. This case, with a T4 tumor invading the vertebral body, a tumor through the neuroforamen, and abutting the spinal cord, would not be possible to have an adequate tumor dose of 60 Gy (which is way above the tolerance dose of the spinal cord) under conventional planning techniques. This was achieved through 1) the use of adaptive radiotherapy based on shrunken tumors, 2) heterogeneous dose prescription to a low small volume of GTV to receive less than the prescribed dose, and 3) isotoxicity principle, i.e., the dose prescription principle is to ensure the dose limits of all organs at risk are met. This approach controlled the tumor while ensuring the safety of the spinal cord. The adaptive radiation therapy, which considered tumor changes, has already been reported to provide excellent local control (15).

It is worth mentioning the post-radiation therapy changes years after the completion of radiotherapy. It is first interesting to see the slow but long-lasting tumor regression along with bone structure recovery after radiotherapy. The mechanism is currently unknown, but some studies suggest that stem cells play a role in repairing the radiation-induced damage (16). Additionally, one should note that post-radiation lung changes in the lung parenchyma on the CT, from inflammation (the first couple of months) to fibrosis (at about 2 years) recovered without any treatment. Studies have reported that the bifunctional fusion protein bintrafusp alfa, which inhibits both TGF-β1 and PD-L1, may play a role in mitigating late-stage radiation therapy-induced lung fibrosis (17). This patient, however, did not receive any anti-inflammatory treatment and had the inflammation and fibrosis almost completely resolved with time after 2 years. This suggests that a watchful waiting approach rather than immediate intervention may be considered for asymptomatic radiographic pneumonitis. However, this observation is derived from a single case and requires further validation before any conclusion can be drawn regarding whether such patients can routinely forego steroid or other anti-inflammatory therapy.

This study is limited by a case report; all findings are hypothesis-generating. Future studies with more cases and well-designed clinical trials are needed. With increasing life expectancy, we shall have more such cases and should pay more attention to the treatment of patients over 90 years. From a societal level, it is also important to identify those who could be cured with chemoradiation therapy exempt from immunotherapy in stage IIIC NSCLC.

## Conclusions

This 90-year-old patient with stage IIIC squamous cell lung cancer has been cured with concurrent chemoradiation therapy without adjuvant immunotherapy. We have learned the following: 1) Carefully selected, fit extremely elderly patients may tolerate and benefit from definitive CCRT. 2) T4 cancers abutting the spinal cord can be treated to a definitive dose of radiotherapy without exceeding the safe limit of the spinal cord. 3) T4 cancer-related destructive bone can be recovered after tumor control and radiographic radiation pneumonitis can be self-resolved without steroid treatment. Future prospective studies are needed to validate the above observations; in particular, biomarker studies identifying patients who do not require adjuvant immunotherapy would have a significant impact on the patients and the society.

## Data Availability

The datasets presented in this article are not readily available because they contain identifiable individual‑level information from de‑identified clinical cases, which cannot be publicly released to comply with institutional ethics requirements and protect patient confidentiality. Requests to access the datasets should be directed to the corresponding author.
